# Supplementary material for: Complexes of tubulin oligomers and tau form a viscoelastic intervening network cross-bridging microtubules into bundles
Source: Nat Commun. 2024 Mar 15;15:2362. doi: 10.1038/s41467-024-46438-x (PMC10943092; doi:10.1038/s41467-024-46438-x)
Supplement: Supplementary file 1 — Supplementary Information [file 41467_2024_46438_MOESM1_ESM.pdf]

Supplementary Information

Complexes of tubulin oligomers and tau form a viscoelastic intervening network cross-bridging microtubules into bundles

Phillip Kohl, Chaeyeon Song, Bretton Fletcher, Rebecca L. Best, Christine Tchounwou, Ximena Garcia, Peter J. Chung, Herbert P. Miller, Leslie Wilson, Myung Chul Choi, Youli Li, Stuart C. Feinstein, and Cyrus R. Safinya

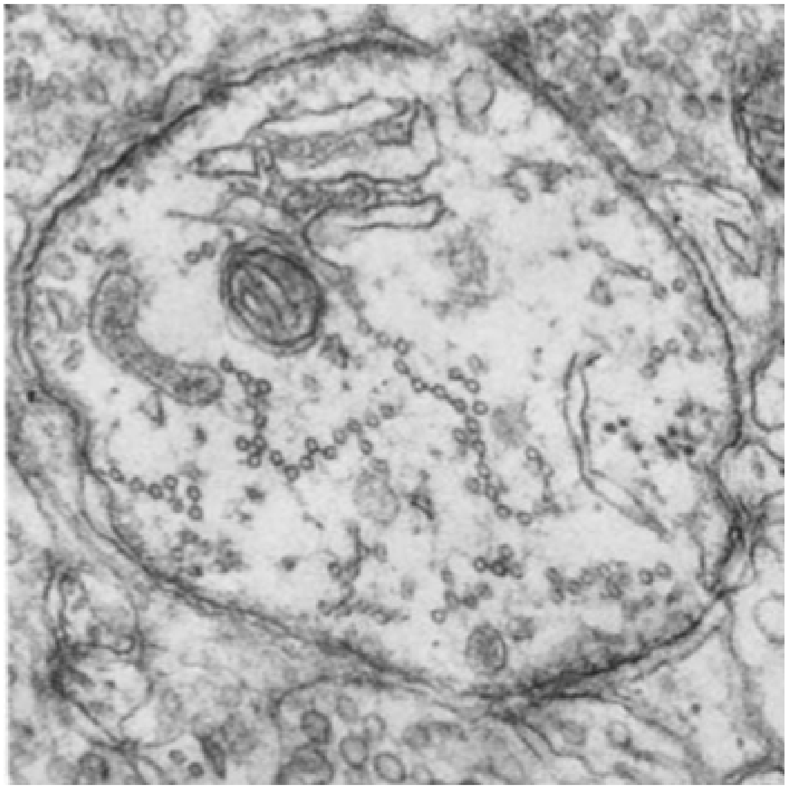

Supplementary Figure 1. Prior electron microscopy revealed linear microtubule bundles (microtubule fascicles) in vivo in the axon initial segment. Linear microtubule bundles in the axon initial segment viewed in a transverse section of a rat cerebral cortex. X 50,000. Adapted from (1), Fig. 6. The diameter of the microtubules is of order 25 nm. Reprinted from ref. [15] with permission from Rockefeller University Press.

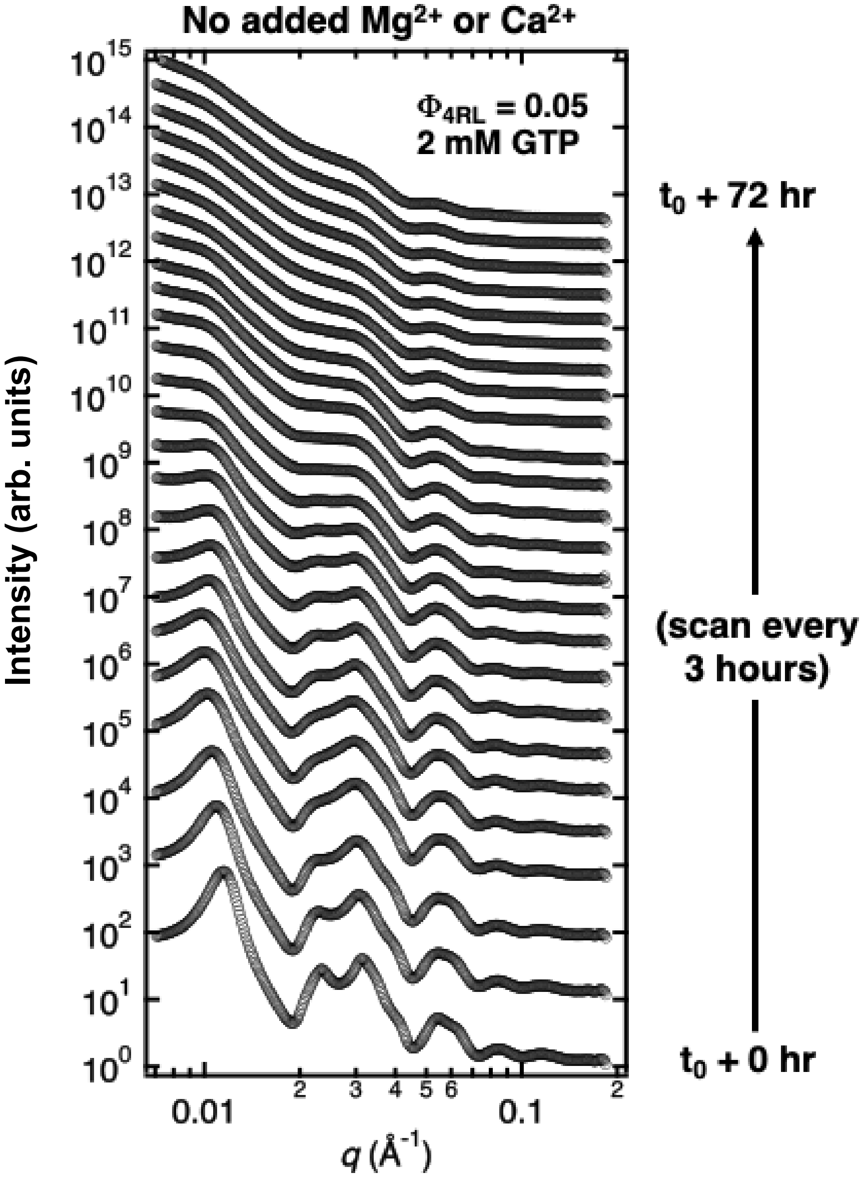

Supplementary Figure 2. Time- dependent synchrotron SAXS data reveals the stability of the wide-spacing (B_{ws}) microtubule bundle state in the absence of added divalent cations. SAXS profiles are of tubulin/tau/GTP mixtures at 37°C and 4RL-tau to tubulin-dimer molar ratio $\Phi_{4RL} = 0.05$. t_0 corresponds to the short time point after sample preparation when the initial SAXS measurement was performed. Azimuthally averaged synchrotron SAXS data (open circles) with increasing time. SAXS scans are offset for clarity. The B_{ws} was stable over the duration of the experiment (72 hours) and only shows a slow broadening and decrease in intensity of the peaks with increasing time due to ongoing suppressed MT dynamic instability in the presence of tau. Despite gradual MT depolymerization over time, scattering from tubulin rings is absent at all time points.

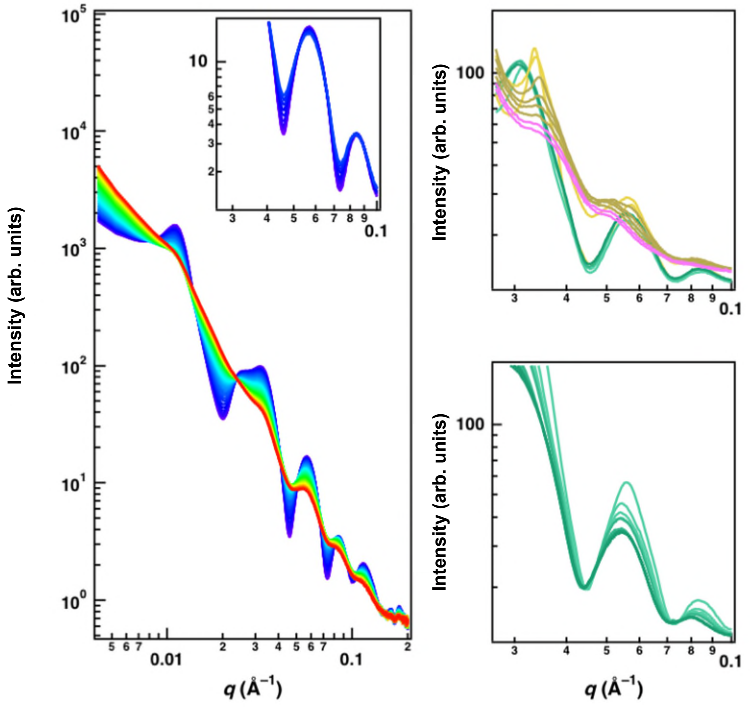

Supplementary Figure 3. Time-dependent SAXS data shows that radiation damage produces distinct scattering features not observed in experimental conditions. (Left) Raw SAXS data was collected using 1-second exposures every two seconds for a period of two minutes. At initial and early timepoints (purple to blue curves), features of the B_{ws} state are prevalent, whereas scattering from later exposures (green to red curves) show increased scattering at low q values and decreased scattering from bundled MTs. Inset shows the first 11 exposures and highlights the continuous increase in scattering at q values associated with minima from the MT form factor. (Right) Zoomed-in raw SAXS data (from Figure 6) from samples containing 1.8 mM and 0.6 mM Mg^{2+} (top and bottom, respectively) show the distinct features of samples that do and do not transition from the B_{ws} state to the B_{int} state, respectively. Specifically, scattering at MT form factor minima does not increase while the B_{ws} is the dominant structural state, but scattering at these form factor minima increase sharply upon transition to the B_{int} state. Following the transition to the B_{int} state, further increases in scattering at FF minima is minimal. Data from Figure 6 was collected as described throughout the main text, with 1-second exposures every 3 hours. The distinguishing features of the intentionally irradiated sample in A are not observed in the experiments reported in the main text.

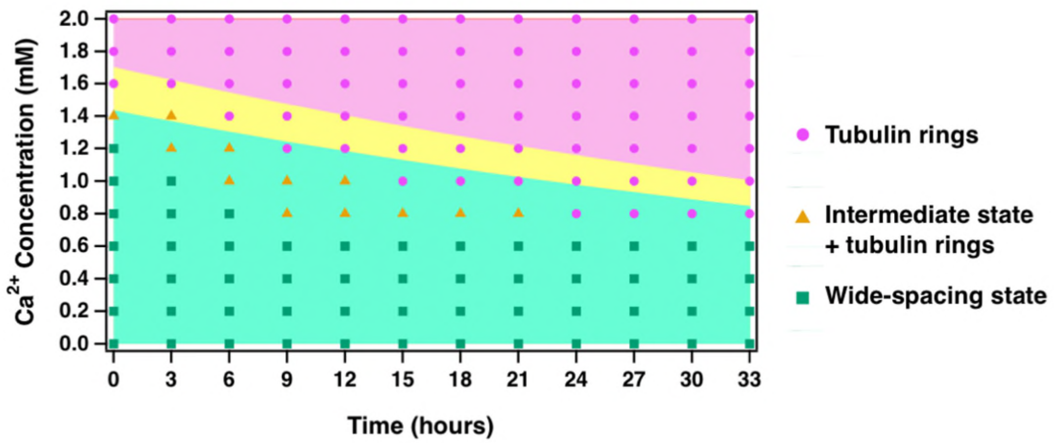

Supplementary Figure 4. Kinetic phase diagram comparison for added $CaCl_2$ with different batches of bovine tubulin. Kinetic phase diagram indicates the structural state of each sample as a function of $CaCl_2$ concentration and time. Markers markers indicate the structural state of one batch of tubulin, while the background color-fill shows the kinetic phase diagram from Figure 3, using a separate batch of tubulin. Tubulin protein was purified from separate bovine brains and produced similar trends in the stability of each structural state.

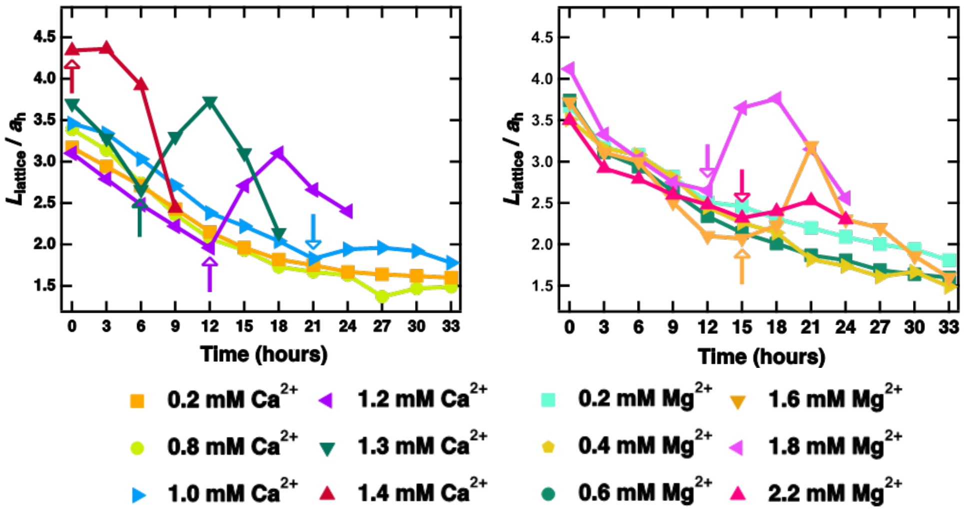

Supplementary Figure 5. Change in the average domain size of the hexagonal lattice, upon transitioning from the wide-spacing (B_{ws}) to the intermediate (B_{int}) MT bundle state.

Time “0” on the x-axis corresponds to the short time point before SAXS data was taken right after sample preparation (referred to as t_0 in figures 2,3). Plots of fitted domain size (L_{domain}) normalized by the lattice parameter (a_h) as a function of time for the SAXS data shown in figures 2 and 3 for the Ca^{2+} (left) and Mg^{2+} (right) series, respectively. Some data points omitted for clarity. Arrows indicate the latest time point the wide-spacing (B_{ws}) state is observed as the sample transitions to the intermediate B_{int} state and domain sizes increase.

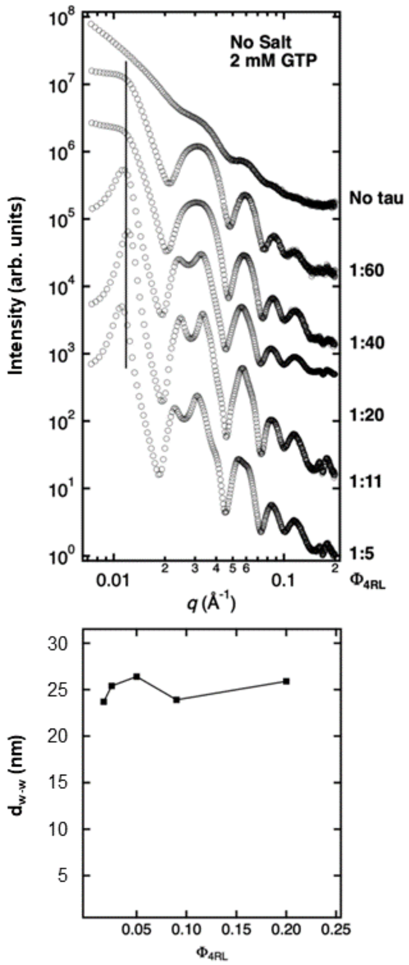

Supplementary Figure 6. Synchrotron SAXS data reveal the wall-to-wall distance of bundled microtubules is not dependent on 4RL-Tau to tubulin-dimer molar ratio. SAXS profiles are of tubulin/tau/GTP mixtures at 37°C at standard buffer conditions with increasing Φ_{4RL} at t_0. Plots of fitted wall-to-wall distance ($d_{w\text{-}w}$) for the corresponding SAXS data highlight the lack of change in $d_{w\text{-}w}$ with increasing Φ_{4RL}. (Top) Azimuthally averaged synchrotron SAXS data (open circles) with decreasing Φ_{4RL}. SAXS scans are offset for clarity. The location of the (1,0) peak, $q_{1,0}$, which is used to measure the center-to-center distance between microtubules is not dependent on Φ_{4RL}. (Bottom) Plots of fitted wall-to-wall spacings ($d_{w\text{-}w}$) as a function of Φ_{4RL} of the SAXS data shown above.

Supplementary Table 1. Reporting Table for SAXS data acquisition, data, analysis, and modelling.

Reporting for tabulating essential SAS data acquisition, sample details, data analysis, modelling fitting and software used.

(*a*) Sample details		
	Tubulin	Tau
Organism	Bovine	Expressed from pDNA containing human MAP Tau gene
Source	Bovine Brain, from slaughterhouse	Human tau plasmid expressed in E. Coli
Description - sequence (including tags) + bound ligands/modifications, etc.	Mixed isoforms of alpha beta tubulin as purified from bovine brain	P10636
Extinction coefficient	N/A. UV absorption coefficient measurement was not needed for the analysis.	N/A. UV absorption coefficient measurement was not needed for the analysis.
Partial specific volume	0.97 cc/g (based SAXS diffraction measurement assuming average protein density of 1.27 g/cc)	0.97 cc/g (based SAXS diffraction measurement assuming average protein density of 1.27 g/cc)
Solute scattering lengths	0.42 e/A^3	0.42 e/A^3
Solvent scattering lengths	0.33 e/A^3	0.33 e/A^3
Scattering contrast	0.089 e/A^3	0.089 e/A^3
M from chemical composition	100 kDa	45.9 kDa
For SEC-SAS, loading volume/concentration, flow rate	N/A. Not SEC-SAXS experiment.	N/A. Not SEC-SAXS experiment
Concentration (range/values) measured and method	N/A. Not SEC-SAXS experiment.	N/A. Not SEC-SAXS experiment.
Solvent details	PEM50, 2mM GTP	PEM50, 2mM GTP

(*b*) SAXS data collection parameters	
Source	SSRL be2mline 4-1
Wavelength	1.3776 A
Beam geometry	0.2 x 0.2 mm square
Sample to detector distance	3530.3
q-measurement range (Å^{-1} or nm^{-1})	0.0035-0.21A
Absolute scaling method	All data reported are in arbitrary units and data was not scaled onto an absolute scale.
Method for monitoring radiation damage, X-ray dose where relevant	Shown in Supplementary Figure 3. To test the effects of radiation damage test samples were prepared and exposed to 1 second of synchrotron radiation 60 times. Data showed that for the exposures under 10 seconds minimal radiation damage was observed.

Exposure time, number of exposures	Each scattering profile shown is from a single 1 second exposure.
Sample configuration including path length and flow rate where relevant	N/A. All samples were loaded and sealed into capillaries. No flow experiments were performed.
Sample temperature	Unless otherwise noted, all samples were run at 37.0 C.

(*c*) Software employed for SAXS data reduction, analysis and interpretation

SAXS data reduction software	Nika SAS
Extinction coefficient	N/A. UV absorption coefficient measurement was not needed for the analysis.
Calculation of $\Delta\rho$	$\Delta\rho = 0.089$ e/A^3
Basic analyses: Guinier, *P*(*r*), Porod volume, volume of correlation	Scattering from disordered tubulin-tau fractions within the mixture is modelled with a combined Guinier-Porod (methods). Ordered tubulin-tau structures are modelled by the Fourier transform of theoretical tubulin-tau lattice, including form and structure factors. Results for Guinier-Porod values are given in the 'source data' file. We note that because tubulin exists in multiple conformations, and because its scattering contribution is much weaker than the bundled microtubule state, the Guinier-Porod results bear little relevance in this particular study.
Shape/bead modelling	N/A. Shape/Bead modelling was not used in this study.
Atomic structure modelling (homology, rigid body, ensemble)	N/A. Atomic modelling was not used in this study.
3D graphic model representations	The 3D models presented in this study were drawn using Blender.

(*d*) Structural parameters

Guinier Analysis	
I(0)	N/A. Experiments were not normalized onto an absolute scale.
R_g	~15 nm, results for each fit are given in the source data file.
qR_g range	~0.6-22.50
Quality-of-fit parameter (with definition)	$\chi^2 < 2$ for all fits
M from *I*(0) (ratio to expected value)	N/A. Experiments were not normalized onto an absolute scale.
P(*r*) analysis	P(r) analysis was not performed on scattering profiles.
I(0)	N/A

R_g	N/A	
d_{max}	N/A	
q range	N/A	
Quality-of-fit parameter (with definition)	N/A	
M from $I(0)$ (ratio to expected value)	N/A	
V_P and/or V_c	N/A	
(*e*) Shape modelling results		
	Bundled MT Phase	Ring Phase
q range for fitting	0.004-0.07	0.004-0.15
Symmetry/anisotropy assumptions	P6mm	none
χ^2 range value/range, other quality of fit parameters	<3 for all fits	<1 for all fits
Adjustable parameters in the model fit	Up to 28 (including 12 for peak amplitudes, 1 for first peak position, 3 for first 3 peak widths, 1 for inner radius of MT, see Methods), however, many of these parameters were held depending on the identified phase, fitted-q range, or time point of the sample (Methods). Results for all fitting parameters can be seen in the Source Data file.	Up to 10, 7 for the unified fit equation and 3 for modelling of the tubulin ring (ring length, radius, and scattering amplitude).
Model precision/resolution	~2A	~2A
R_g values for multiple phase shape models	90.4 (for MT hollow cross-section)	133.7
(*f*) Atomistic modelling		
	N/A. Atomistic modelling was not used	
q range for fitting	N/A	
Symmetry assumptions	N/A	
Any measures of model precision	N/A	
χ^2 range value/range, other quality of fit parameters	N/A	
Adjustable parameters in the model fit	N/A	
Domain/subunit contacts and regions of presumed flexibility for atomistic modelling	N/A	
(*g*) Data and model deposition IDs		
