# Supplementary material for: Complexes of tubulin oligomers and tau form a viscoelastic intervening network cross-bridging microtubules into bundles
Source: Nat Commun. 2024 Mar 15;15:2362. doi: 10.1038/s41467-024-46438-x (PMC10943092; doi:10.1038/s41467-024-46438-x)
Supplement: Supplementary file 3 — Description of Additional Supplementary Files [file 41467_2024_46438_MOESM3_ESM.pdf]

Description of Additional Supplementary Files

File Name: Supplementary Code 1
Description: Code for modeling of MT bundles and tubulin oligomers.
