# Supplementary material for: Complexes of tubulin oligomers and tau form a viscoelastic intervening network cross-bridging microtubules into bundles
Source: Nat Commun. 2024 Mar 15;15:2362. doi: 10.1038/s41467-024-46438-x (PMC10943092; doi:10.1038/s41467-024-46438-x)
Supplement: Supplementary file 4 — Supplementary Code 1 [file 41467_2024_46438_MOESM4_ESM.pdf]

Supplementary Code: CPLOT Fitting Function

```
#define VERSION 5
#define NUM_XS 1 /* Num of independent variables */
#define MAXPAR 28 /* Num of parameters */
#define MAXPTS 500 /* Most points for fitting */
#define MAXPLT 500 /* Most for pg ps md mr sA */
#include <math.h>
#include <p_fitsize.h>

char *title = "Fits first 12 peaks from hexagonally bound MTs, single-ring tubulin FF, unified fit
BG";

char *comment = "";

void
setup() {
  set_prompt(0, "LINE");
  set_prompt(1, "line");
}

struct init_4 initial[] = {

/* Name         Deriv?  Fit?  Initial  Limit?  Low  High  */
{"G",      0,    1,           0,    1,     0,    1}, /* Coefficient for BG Guiner exp*/
{"Rg",     0,    0,      19000.0,    3,    10,20000}, /* Rg for Guiner exp */
{"B1",     0,    1,      .05514,    1,     0,    0}, /* Coefficient for the low-q Porod region (mass
fractal)*/
{"Pwr1",   0,    1,       3.886,    3,     1,    4}, /* Power law for mass fractal*/
{"B2",     0,    1,   .000530184,    1,     0,    0}, /* Coefficient for the high-q Porod region (surface
fractal)*/
{"Pwr2",   0,    1,      1.82616,    3,     1,    4}, /* Power law for surface fractal*/
{"y0",     0,    0,      0.42324,    3,     0,   15}, /* flat BG scattering */
{"Aring",  0,    0,        0e-9,    1,     0,    0}, /* Coefficient for Ring FF */
{"Lring",  0,    0,        38.5,    1,     0,    0}, /* L = length of ring */
{"W",      0,    0,          49,    3,    30,   50}, /* W = width of tubulin */
{"Rring",  0,    0,      162.98,    3,    75,  220}, /* Rring = inner radius of the tubulin ring */
{"q10",            0,       1,     .0121360,    3,  .008,  .02}, /* q10 = q of Imax */
{"Rin",    0,    0,       94.41,    3,    90,  180}, /* Rin = inner radius of MT */
{"P1",     0,    1,  1.25645e-10,    3, 1e-20, 1e-6}, /* coefficient for peak 1 */
{"K",      0,    1,   0.00308035,    3, 0.001, 0.01}, /* K = 1.554*HWHM at peak 1*/
{"P2",     0,    1,  1.24528e-10,    3, 1e-20, 1e-6}, /* coefficient for peak 2  */
{"K2",     0,    0,           0.0035,    3, 0.001, 0.01}, /* K2 = 1.554*HWHM at peak 2*/
{"P3",     0,    0,     1.00e-11,    3, 1e-20, 1e-8}, /* coefficient for peak 3 */
{"K3",     0,    0,       0.0055,    3, 0.001, 0.01}, /* K3 = 1.554*HWHM at peak 3 */
{"P4",     0,    0,     1.01e-11,    3, 1e-20, 1e-8}, /* coefficient for peak 4 */
```

```
{"P5",    0,   0,    1.00e-11,   3, 1e-20, 1e-8}, /* coefficient for peak 5 */
{"P6",    0,   0,    1.00e-11,   3, 1e-20, 1e-8}, /* coefficient for peak 6 */
{"P7",    0,   0,    1.00e-11,   3, 1e-20, 1e-8}, /* coefficient for peak 7 */
{"P8",    0,   0,    1.00e-11,   3, 1e-20, 1e-8}, /* coefficient for peak 8 */
{"P9",    0,   0,    1.00e-11,   3, 1e-20, 1e-8}, /* coefficient for peak 9 */
{"P10",   0,   0,    1.00e-11,   3, 1e-20, 1e-8}, /* coefficient for peak 10 */
{"P11",   0,   0,    1.00e-11,   3, 1e-20, 1e-8}, /* coefficient for peak 11 */
{"P12",   0,   0,    1.00e-11,   3, 1e-20, 1e-8}, /* coefficient for peak 12 */
};

/* define the parameters */

#define G      fpar[0].p_b
#define fG     fpar[0].p_fit
#define dG     fpar[0].p_p

#define Rg      fpar[1].p_b
#define fRg     fpar[1].p_fit
#define dRg     fpar[1].p_p

#define B1      fpar[2].p_b
#define fB1     fpar[2].p_fit
#define dB1     fpar[2].p_p

#define Pwr1    fpar[3].p_b
#define fPwr1   fpar[3].p_fit
#define dPwr1   fpar[3].p_p

#define B2     fpar[4].p_b
#define fB2    fpar[4].p_fit
#define dB2    fpar[4].p_p

#define Pwr2      fpar[5].p_b
#define fPwr2     fpar[5].p_fit
#define dPwr2     fpar[5].p_p

#define y0      fpar[6].p_b
#define fy0     fpar[6].p_fit
#define dy0     fpar[6].p_p

#define Aring      fpar[7].p_b
#define fAring     fpar[7].p_fit
#define dAring     fpar[7].p_p

#define Lring  fpar[8].p_b
#define fLring fpar[8].p_fit
```

```
#define dLringfpar[8].p_p

#define W      fpar[9].p_b
#define fW     fpar[9].p_fit
#define dW     fpar[9].p_p

#define Rring      fpar[10].p_b
#define fRring     fpar[10].p_fit
#define dRring     fpar[10].p_p

#define q10      fpar[11].p_b
#define fq10     fpar[11].p_fit
#define d10     fpar[11].p_p

#define Rin    fpar[12].p_b
#define fRin   fpar[12].p_fit
#define dRin   fpar[12].p_p

#define P1     fpar[13].p_b
#define fP1    fpar[13].p_fit
#define dP1    fpar[13].p_p

#define K      fpar[14].p_b
#define fK     fpar[14].p_fit
#define dK     fpar[15].p_p

#define P2     fpar[15].p_b
#define fP2    fpar[15].p_fit
#define dP2    fpar[15].p_p

#define K2     fpar[16].p_b
#define fK2    fpar[16].p_fit
#define dK2    fpar[16].p_p

#define P3     fpar[17].p_b
#define fP3    fpar[17].p_fit
#define dP3    fpar[17].p_p

#define K3     fpar[18].p_b
#define fK3    fpar[18].p_fit
#define dK3    fpar[18].p_p

#define P4     fpar[19].p_b
#define fP4    fpar[19].p_fit
#define dP4    fpar[19].p_p
```

```
#define P5     fpar[20].p_b
#define fP5    fpar[20].p_fit
#define dP5    fpar[20].p_p

#define P6     fpar[21].p_b
#define fP6    fpar[21].p_fit
#define dP6    fpar[21].p_p

#define P7     fpar[22].p_b
#define fP7    fpar[22].p_fit
#define dP7    fpar[22].p_p

#define P8     fpar[23].p_b
#define fP8    fpar[23].p_fit
#define dP8    fpar[23].p_p

#define P9     fpar[24].p_b
#define fP9    fpar[24].p_fit
#define dP9    fpar[24].p_p

#define P10    fpar[25].p_b
#define fP10   fpar[25].p_fit
#define dP10   fpar[25].p_p

#define P11    fpar[26].p_b
#define fP11   fpar[26].p_fit
#define dP11   fpar[26].p_p

#define P12    fpar[27].p_b
#define fP12   fpar[27].p_fit
#define dP12   fpar[27].p_p


/*define the independent variables */

#define Q       (M_flag? Make_x[0]:dp->d_xx[0])

double model(int deriv_flag)
{
        double q, Sfit, j, FFring=0, FFmt, SFmt, x, wt, L=20000, Wmt = 49;
        int i;
        q = Q;
        wt = M_PI/2048;
        for (i=1;i<2049;i++) {
                x = cos((2*i-1)*M_PI/(2*2048));
```

```
			FFmt = pow(sin(q*x*L/2)/(q*q*x)*((Rin+Wmt)*j1(q*sqrt(1-x*x)*(Rin+Wmt))-
Rin*j1(q*sqrt(1-x*x)*Rin)),2)/sqrt(1-x*x);
			SFmt = pow(P1/(K*K+(q*sqrt(1-x*x)-q10)*(q*sqrt(1-x*x)-q10)),
2)+pow(P2/(K2*K2+(q*sqrt(1-x*x)-sqrt(3)*q10)*(q*sqrt(1-x*x)-sqrt(3)*q10)),
2)+pow(P3/(pow(K3,2)+(q*sqrt(1-x*x)-2*q10)*(q*sqrt(1-x*x)-2*q10)),
2)+pow(P4/(pow(K3,2)+(q*sqrt(1-x*x)-sqrt(7)*q10)*(q*sqrt(1-x*x)-sqrt(7)*q10)),
2)+pow(P5/(pow(K3,2)+(q*sqrt(1-x*x)-3*q10)*(q*sqrt(1-x*x)-3*q10)),
2)+pow(P6/(pow(K3,2)+(q*sqrt(1-x*x)-sqrt(12)*q10)*(q*sqrt(1-x*x)-sqrt(12)*q10)),
2)+pow(P7/(pow(K3,2)+(q*sqrt(1-x*x)-sqrt(13)*q10)*(q*sqrt(1-x*x)-sqrt(13)*q10)),
2)+pow(P8/(pow(K3,2)+(q*sqrt(1-x*x)-4*q10)*(q*sqrt(1-x*x)-4*q10)),
2)+pow(P9/(pow(K3,2)+(q*sqrt(1-x*x)-sqrt(19)*q10)*(q*sqrt(1-x*x)-
sqrt(19)*q10)),2)+pow(P10/(pow(K3,2)+(q*sqrt(1-x*x)-sqrt(21)*q10)*(q*sqrt(1-x*x)-
sqrt(21)*q10)), 2)+pow(P11/(pow(K3,2)+(q*sqrt(1-x*x)-5*q10)*(q*sqrt(1-x*x)-5*q10)),
2)+pow(P12/(pow(K3,2)+(q*sqrt(1-x*x)-sqrt(27)*q10)*(q*sqrt(1-x*x)-sqrt(27)*q10)), 2);
			FFring = pow(sin(q*x*Lring/2)/(q*q*x)*((Rring+W)*j1(q*sqrt(1-
x*x)*(Rring+W))-Rring*j1(q*sqrt(1-x*x)*Rring)),2)/sqrt(1-x*x);
			Sfit = Sfit+Aring*FFring+FFmt*SFmt;
		}
		if (deriv_flag) {

		}
		Sfit = Sfit*wt+y0+G*exp(-1*q*q*Rg*Rg/3)+B2*pow(pow(erf(q*Rg/sqrt(6)),
3)/q,Pwr2)+B1*exp(-1*Rg*Rg*q*q/3)*pow(pow(erf(q*1e10/sqrt(6)), 3)/q,Pwr1);
		return(Sfit);
}

int	prefilter(), postfilter();
struct  user_cmds {
	char	c_one;
	char	two;
	int	(*c_func)();
} user_cmds[] = {
	{'n', 'm', postfilter},
	{'m', 'n', prefilter},
	0,
};
prefilter() {
}
postfilter() {
}
```
