# Supplementary material for: Complexes of tubulin oligomers and tau form a viscoelastic intervening network cross-bridging microtubules into bundles
Source: Nat Commun. 2024 Mar 15;15:2362. doi: 10.1038/s41467-024-46438-x (PMC10943092; doi:10.1038/s41467-024-46438-x)
Supplement: Supplementary file 5 — Reporting Summary [file 41467_2024_46438_MOESM5_ESM.pdf]

nature portfolio

Corresponding author(s): Cyrus R. Safinya

Last updated by author(s): Feb 9, 2024

Reporting Summary

Nature Portfolio wishes to improve the reproducibility of the work that we publish. This form provides structure for consistency and transparency in reporting. For further information on Nature Portfolio policies, see our Editorial Policies and the Editorial Policy Checklist.

Statistics

For all statistical analyses, confirm that the following items are present in the figure legend, table legend, main text, or Methods section.

n/a	Confirmed	
☐	☒	The exact sample size (*n*) for each experimental group/condition, given as a discrete number and unit of measurement
☐	☒	A statement on whether measurements were taken from distinct samples or whether the same sample was measured repeatedly
☒	☐	The statistical test(s) used AND whether they are one- or two-sided<br>*Only common tests should be described solely by name; describe more complex techniques in the Methods section.*
☒	☐	A description of all covariates tested
☐	☒	A description of any assumptions or corrections, such as tests of normality and adjustment for multiple comparisons
☐	☒	A full description of the statistical parameters including central tendency (e.g. means) or other basic estimates (e.g. regression coefficient) AND variation (e.g. standard deviation) or associated estimates of uncertainty (e.g. confidence intervals)
☒	☐	For null hypothesis testing, the test statistic (e.g. *F*, *t*, *r*) with confidence intervals, effect sizes, degrees of freedom and *P* value noted<br>*Give P values as exact values whenever suitable.*
☒	☐	For Bayesian analysis, information on the choice of priors and Markov chain Monte Carlo settings
☒	☐	For hierarchical and complex designs, identification of the appropriate level for tests and full reporting of outcomes
☒	☐	Estimates of effect sizes (e.g. Cohen's *d*, Pearson's *r*), indicating how they were calculated

Our web collection on statistics for biologists contains articles on many of the points above.

Software and code

Policy information about availability of computer code

Data collection	The data collection was performed with a beam-line controlled software provided to the users of the Nation Facility at the Stanford Synchrotron Radiation Lightsource (part of Stanford Linear Accelerator Center).
Data analysis	2D scattering images were reduced using Nika (version 1.83). Data analysis involved use of software for nonlinear least squares fitting routines based on the Marquardt algorithm. The software package used was C-PLOT (version 5.0) which is a commercially available package from Certified Scientific Software.

For manuscripts utilizing custom algorithms or software that are central to the research but not yet described in published literature, software must be made available to editors and reviewers. We strongly encourage code deposition in a community repository (e.g. GitHub). See the Nature Portfolio guidelines for submitting code & software for further information.

Data

Policy information about availability of data

All manuscripts must include a data availability statement. This statement should provide the following information, where applicable:

- Accession codes, unique identifiers, or web links for publicly available datasets
- A description of any restrictions on data availability
- For clinical datasets or third party data, please ensure that the statement adheres to our policy

All raw and processed data in the paper is given in the source data file.

Human research participants

Policy information about studies involving human research participants and Sex and Gender in Research.

Reporting on sex and gender	N/A
Population characteristics	N/A
Recruitment	N/A
Ethics oversight	N/A

Note that full information on the approval of the study protocol must also be provided in the manuscript.

Field-specific reporting

Please select the one below that is the best fit for your research. If you are not sure, read the appropriate sections before making your selection.

- [x] Life sciences
- [ ] Behavioural & social sciences
- [ ] Ecological, evolutionary & environmental sciences

For a reference copy of the document with all sections, see nature.com/documents/nr-reporting-summary-flat.pdf

Life sciences study design

All studies must disclose on these points even when the disclosure is negative.

Sample size	The work is a cell free biophysical study of tubulin and tau self-assembly in dissipative (i.e. out-of-equilibrium) reaction mixtures containing GTP at controlled temperatures. For each metal ion concentration, on average between 3 to 5 reaction mixtures were prepared and studied. The data is a result of more than 10 independent synchrotron runs and the Stanford Radiation Lightsource. As X-ray experiments probe the entire population of MTs within a sample, characterization with this technique are not susceptible to biases from small sample size.
Data exclusions	No data was excluded in the statistical analysis of the data.
Replication	The key features in the SAXS data line-shape analysis were highly reproducible in each of the three main phases reported in the study. The features were further reproducible across all protein batches.
Randomization	The randomization process in the reported biophysical study at the assembled macromolecular level is not relevant. This is because of a lack of bias related to the preparation of reaction mixtures contain either magnesium, calcium, or no added salt.
Blinding	The data collection and analysis result from objective measurements of scattering of x-ray photons due to all structural features present in the entire sample. Thus, no blinding was required.

Reporting for specific materials, systems and methods

We require information from authors about some types of materials, experimental systems and methods used in many studies. Here, indicate whether each material, system or method listed is relevant to your study. If you are not sure if a list item applies to your research, read the appropriate section before selecting a response.

Materials & experimental systems

n/a	Involved in the study	
☒	☐	Antibodies
☒	☐	Eukaryotic cell lines
☒	☐	Palaeontology and archaeology
☐	☒	Animals and other organisms
☒	☐	Clinical data
☒	☐	Dual use research of concern

Methods

n/a	Involved in the study	
☒	☐	ChIP-seq
☒	☐	Flow cytometry
☒	☐	MRI-based neuroimaging

Animals and other research organisms

Policy information about studies involving animals; ARRIVE guidelines recommended for reporting animal research, and Sex and Gender in Research

Laboratory animals	The study did not involve laboratory animals.
Wild animals	The study did not involve wild animals.
Reporting on sex	Sex-based analysis were not performed within this study as the proteins used within this study do not differ between sex.
Field-collected samples	No field-collected samples were used in this study.
Ethics oversight	No ethical approval or guidance was necessary as the bovine brain used in this study was purchased from a commercial slaughterhouse.

Note that full information on the approval of the study protocol must also be provided in the manuscript.
